# Genome‑wide association study and genomic prediction for growth traits in yellow-plumage chicken using genotyping-by-sequencing

**DOI:** 10.1186/s12711-021-00672-9

**Published:** 2021-10-27

**Authors:** Ruifei Yang, Zhenqiang Xu, Qi Wang, Di Zhu, Cheng Bian, Jiangli Ren, Zhuolin Huang, Xiaoning Zhu, Zhixin Tian, Yuzhe Wang, Ziqin Jiang, Yiqiang Zhao, Dexiang Zhang, Ning Li, Xiaoxiang Hu

**Affiliations:** 1 State Key Laboratory of Agrobiotechnology, College of Biological Sciences, China Agricultural University, Beijing, China; 2 College of Animal Science and Technology, China Agricultural University, Beijing, China; 3 Wen’s Nanfang Poultry Breeding Co. Ltd, Yunfu, 527400 Guangdong Province China

## Abstract

**Background:**

Growth traits are of great importance for poultry breeding and production and have been the topic of extensive investigation, with many quantitative trait loci (QTL) detected. However, due to their complex genetic background, few causative genes have been confirmed and the underlying molecular mechanisms remain unclear, thus limiting our understanding of QTL and their potential use for the genetic improvement of poultry. Therefore, deciphering the genetic architecture is a promising avenue for optimising genomic prediction strategies and exploiting genomic information for commercial breeding. The objectives of this study were to: (1) conduct a genome-wide association study to identify key genetic factors and explore the polygenicity of chicken growth traits; (2) investigate the efficiency of genomic prediction in broilers; and (3) evaluate genomic predictions that harness genomic features.

**Results:**

We identified five significant QTL, including one on chromosome 4 with major effects and four on chromosomes 1, 2, 17, and 27 with minor effects, accounting for 14.5 to 34.1% and 0.2 to 2.6% of the genomic additive genetic variance, respectively, and 23.3 to 46.7% and 0.6 to 4.5% of the observed predictive accuracy of breeding values, respectively. Further analysis showed that the QTL with minor effects collectively had a considerable influence, reflecting the polygenicity of the genetic background. The accuracy of genomic best linear unbiased predictions (BLUP) was improved by 22.0 to 70.3% compared to that of the conventional pedigree-based BLUP model. The genomic feature BLUP model further improved the observed prediction accuracy by 13.8 to 15.2% compared to the genomic BLUP model.

**Conclusions:**

A major QTL and four minor QTL were identified for growth traits; the remaining variance was due to QTL effects that were too small to be detected. The genomic BLUP and genomic feature BLUP models yielded considerably higher prediction accuracy compared to the pedigree-based BLUP model. This study revealed the polygenicity of growth traits in yellow-plumage chickens and demonstrated that the predictive ability can be greatly improved by using genomic information and related features.

**Supplementary Information:**

The online version contains supplementary material available at 10.1186/s12711-021-00672-9.

## Background

Growth traits of chickens are well known for their genetic architectural complexity. Since the release of the chicken genome assembly [1], many researchers have begun to study economic traits for chickens by combining large-scale genomic and phenotypic information. In 2006, Park et al. used an advanced intercross line (AIL) population from two chicken lineages to demonstrate the presence of quantitative trait loci (QTL) on multiple chromosomes [2], providing a glimpse into the polygenicity of chicken growth traits. Thereafter, Johansson et al. [3] estimated that the genetic variation of chicken growth traits was the result of more than 100 genome-wide loci. Two other studies further revealed that genome-wide loci under selection accounted for at least 40% of the phenotypic variation in body weight at 56 days of age in an intercross chicken line, suggesting that bidirectional selection for growth traits acts on many genetic loci, rather than on only a few loci [4, 5].

In 2001, Meuwissen et al. [6] proposed a genomic prediction (GP) method that uses genetic markers across the whole genome. Recently, this method has been extensively studied and applied to many species [7–10]. Several studies using GP have reported improved predictive capacity for chicken production and laying traits [11–13], and GP is currently widely applied in poultry breeding [14]. With the development of next-generation sequencing technologies (NGS), large-scale and low-cost genotyping has recently become available. Sequencing provides the ability to further understand the polygenicity of chicken growth traits and offers opportunities to further improve the accuracy of GP.

The Chicken QTL Database (https://www.animalgenome.org/cgi-bin/QTLdb/GG/index) contains information on 3828 records related to chicken growth traits [15]. Regardless of their genetic background, the loci associated with growth traits in chickens occur throughout the genome. Although growth traits are well known for their polygenicity [16–18], further investigations are needed to identify how and how many genetic loci, specifically, affect chicken growth traits. Such information is essential for identifying genomic features that contribute to improving the performance of genomic prediction methods.

In this study, we used a genotyping-by-sequencing (GBS) strategy to conduct a large-scale genomic analysis of the nucleus breeding population of commercial yellow-plumage chickens. Based on 151,519 autosomal single-nucleotide polymorphisms (SNPs) located along the genome of 6359 chickens, we identified multiple QTL that affect growth traits with major or minor effects. We performed genomic evaluation and estimated the contribution of each locus to the accuracy of genomic predictions. Thus, the objectives of this study were to: (1) conduct a genome-wide association study (GWAS) in yellow-plumage chickens to identify key genetic factors and explore the polygenicity of growth traits in chickens; (2) investigate the accuracy of GP in this population; and (3) evaluate genomic predictions that harness genomic features.

## Methods

### Animals, phenotypes, genotypes, and population structure

The animal and phenotypic data used for this study were obtained from a commercial nucleus breeding population of yellow-plumage chickens. Two generations (8 and 9) of chickens hatched in six batches from 2018 to 2019 (2769 males and 5353 females) were generated, managed, and penned in a single nucleus breeding farm. Approximately 1400 candidate roosters and ~ 2800 candidate hens (male:female = 1:2) were available in each generation, and ~ 50 roosters and ~ 750 hens were selected for breeding the next generation; the main selective trait was feed conversion ratio (FCR), with a selection pressure of 1:20 for males and 1:3–4 for females.

All birds were fed ad libitum, following the feeding management procedure of the company during the brooding stage (1–35 days) and the growing stage (36–84 days). During the brooding stage, the birds were raised in a closed brooding area. They were then transferred to pens with vertical ventilation systems for the growing stage, where the males and females were separated and penned at different sides of the building. Each pen had 17 feeding stations and five hanging water fountains. Birds with physical maldevelopment or deformities were eliminated. During the brooding stage birds were fed a diet containing 2837 kcal metabolic energy/kg and 200 g crude protein/kg, before switching to a high-energy diet containing 2900 kcal/kg and 190 g/kg. Daily feed intake of each bird was recorded throughout the feeding trial from 42 to 84 days of age by subtracting the food remaining in the afternoon from the initial mass recorded in the morning. Body weight was recorded at 42 and 84 days of age (denoted as 42-day-old weight (DW) and 84 DW, respectively); FCR was calculated by dividing the total feed intake during the 42- to 84-day period by the total weight gained during that same period, and the average daily gain (ADG) was calculated by dividing the total weight gained during the 42- to 84-day period by 42 days. In total, 8122 body weight phenotypes (42 DW, 84 DW, and ADG) and 8115 FCR phenotypes were recorded. Basic statistics for these traits are in Table 1. All phenotypic values followed an approximately bell-shaped distribution (see Additional file 1: Figure S1).

**Table 1:** Basic statistics of the growth-related phenotypes

Phenotype	Sex	Number of records	Mean ± SD	Median
42 DW (g)	Male	2769	853.9 ± 63.3	860
	Female	5353	731.3 ± 51.9	730
84 DW (g)	Male	2769	2472.0 ± 160.8	2480
	Female	5353	2005.9 ± 155.0	2005
FCR	Male	2767	3.06 ± 0.23	3.05
	Female	5348	3.55 ± 0.33	3.51
ADG (g/day)	Male	2769	38.5 ± 3.5	38.6
	Female	5353	30.4 ± 3.2	30.4

*DW* day-old weight, *FCR* feed conversion ratio, *ADG* average daily gain, *SD* standard deviation

From the full set of 8122 birds, 6684 were sequenced using the GBS method, of which the genotypes were successfully obtained for 6359 individuals (1870 from generation 8 and 4489 from generation 9). Genomic DNA was extracted from blood samples and quantified using a Qubit 2.0 Fluorometer. DNA concentrations were normalised to 200 ng in each well of 96-well plates. Two-enzyme GBS was used, with the enzymes EcoRI and MseI. A set of 96 forward barcoded adapters with an EcoRI overhang was designed using the GBS Barcode Generator (http://www.deenabio.com/services/gbs-adapters) and a reverse adapter with an MseI overhang was designed according to Wang et al. [19]. DNA samples were digested with EcoRI and MseI and then ligated to the designed adapters. Following adapter ligation, the samples were pooled using 96-plex molecular barcoding and then size-selected via purification with AMPure XP Beads (VAHTS). The insert sizes of the libraries were detected using the Agilent 2100 Bioanalyzer. The purified libraries were obtained using an MGIEasy Circularization Kit, amplified by PCR and sequenced on an MGISeq2000 by PE100 sequencing. The SNP genotypes were determined according to the pipeline implemented in Tassel 5.0, with default parameters [20]. We used the genome version of *Gallus gallus* 6.0 as a reference. The average genome sequencing depth reached 0.78 × /bird. After SNP detection, data on 6684 individuals with genotypes on 1,994,350 SNPs were obtained. Beagle 5.0 was used to impute missing SNP genotypes [21] after SNP filtering quality control. The quality control procedure was based on whether the autosomal SNPs had a minor allele frequency higher than 5%, a genotype quality score higher than 98, with a missing rate less than 60%, and whether they passed the Hardy–Weinberg equilibrium test at *p* ≥ 1e − 4. We also removed individuals with a SNP missing rate higher than 30%. Finally, 1,842,831 low-quality SNPs and 325 birds with high SNP missing rates were removed, and 6359 individuals with 151,519 autosomal SNPs were retained with a marker density of one SNP per 6.34 kb. SNPs were uniformly distributed along the entire genome (see Additional file 1: Figure S2). Furthermore, principal component analysis based on a genome-wide complex trait analysis (GCTA) [22] of all birds showed no distinct population stratification (see Additional file 1: Figure S3), demonstrating that population stratification would not influence the GWAS or GS analyses.

### Genome-wide association study

A mixed linear model approach was used for the single-SNP GWAS, as implemented in the GCTA software [22]. The mixed linear model for the GWAS was:

$${\mathbf{y}}={\mathbf{Xb}}+{\mathbf{Zg}}+{\mathbf{e}},$$

where ${\mathbf{y}}$ is the vector of phenotypic observations; ${\mathbf{b}}$ is the vector of fixed effects, including batch–sex effects and genotype at the SNP, which were fitted as covariates; ${\mathbf{X}}$ is the incidence matrix for ${\mathbf{b}}$; ${\mathbf{g}}$ is the vector of random polygenic additive effects, i.e. the accumulated effect of all SNPs, as captured by the genomic relationship matrix **(**GRM); ${\mathbf{Z}}$ is an incidence matrix allocating polygenic effects to phenotypic observations; and ${\mathbf{e}}$ is the vector of random residuals. The GRM was calculated using all SNPs, as implemented in the GCTA software, and the effect of each SNP was evaluated separately. The whole population data used here were not used for further GP. Bonferroni correction was applied to correct for multiple testing across the genome and compensate for the number of markers, with the threshold set at 0.05/151,519 = 3.30e − 7. The candidate QTL interval was based on the level of linkage disequilibrium (LD) between the most significant SNP and its neighbours, with the boundaries based on LD dropping below 0.2. The LD between SNPs was determined using the PLINK software [23] with options “–ld-window-kb 20,000 –ld-snp p –ld-window 15,000”, where p represents the most significant SNP.

### Mixed model for the best linear unbiased prediction

Two generations of birds were used to construct the relationship matrix. The mixed model for genomic best linear unbiased prediction (GBLUP) was the same as that used for GWAS, except that SNP genotype was removed as a fixed effect. In this model:where ${\mathbf{I}}$ denotes the identity matrix,  is the additive genetic variance, and  is the residual variance. The GRM, $\mathbf{G}$, was calculated as implemented in the GVCBLUP package, and the variance components were estimated using genomic restricted maximum likelihood estimation (GREML) via the GREML_CE programme in the GVCBLUP package [25]. Genomic heritability was defined as:

The GBLUP of individual genetic values in the reference and validation samples were calculated during the last GREML iteration. The heritability and effect of each SNP were also computed using the GREML_CE programme.

To further improve the GP model by harnessing genomic features, we used a two-GRM GBLUP model (GFBLUP) to perform genomic evaluations. First, we defined a population for the GWAS (N = 1500) by random sampling from 6359 birds; this population was used to identify significant SNPs and QTL (i.e. the discovery population). Then, we separated the significant SNPs obtained from the GWAS from the 151,519-SNP dataset and generated two GRM for the birds not used in GWAS analysis (N = 4859) (see Additional file 1: Figure S4). One GRM was based on significant SNPs and the other was based on the remaining SNPs. GBLUP analysis was performed using the MTG2 software [26]. The GFBLUP model [27] is described as follows:

$${\text{GFBLUP model}}: \mathbf{y}={\mathbf{X}}_{\mathbf{b}}\mathbf{b}+\mathbf{Z}\mathbf{s}+\mathbf{Z}\mathbf{a}+\mathbf{e},$$

where $\mathbf{s}$ and $\mathbf{a}$ are the vectors of additive genetic values based on significant and non-significant SNPs, respectively, ${\mathbf{G}}_{\mathbf{s}}$ and ${\mathbf{G}}_{\mathbf{a}}$ are the associated GRM and  and  of the associated additive genetic variances; and  is the residual variance.

For pedigree-based BLUP (ABLUP), the GRM of the GBLUP model was replaced by the pedigree-based additive relationship matrix $\mathbf{A}$, calculated using two generations of pedigree-based relationships with the BLUPF90 package [24]. The last round of iteration for  and  computed using the AIREMLF90 programme in the BLUPF90 package was used to predict the validation data.

### Cross-validation

A tenfold cross-validation was conducted to evaluate the predictive ability [28, 29] of the different models. The 4859 genotyped birds remaining after selection of the discovery population for GWAS were randomly divided into ten validation datasets. For each run, nine partitions were treated as the reference group, whereas the remaining one was used for validation. Phenotypic observations in the validation dataset were omitted from the ABLUP, GBLUP, and GFBLUP calculations (see Additional file 1: Figure S4).

To estimate the contribution of QTL regions to heritability and prediction accuracy, we used GBLUP and GFBLUP models to estimate heritability and evaluate prediction accuracy using all the birds (N = 6359). Similarly to the above steps, we randomly divided these birds into ten validation datasets, and for each run, nine partitions were treated as the reference group, whereas the remaining one was used for validation. Phenotypic observations in the validation dataset were omitted from the GBLUP and GFBLUP models.

To evaluate the influence of the reference population size on the prediction accuracy of GBLUP, we randomly selected different subsets of the population from the full set of 6359 birds (550; 1100; 1650; 2200; 2750; 3300; 3850; 4400; 4950; 5500; 6050; and the full set of 6359). For each subset, data were randomly partitioned into 11 equal portions: for each analysis ten portions were used as the reference group for the GBLUP model (i.e. 500; 1000; 1500; 2000; 2500; 3000; 3500; 4000; 4500; 5000; 5500; and 5781, respectively), while the remaining portion was used for validation (i.e. 50; 100; 150; 200; 250; 300; 350; 400; 450; 500; 550; and 578, respectively). For example, when the size of the population was 550, 500 birds were randomly subsampled as the reference set, and the remaining 50 birds were used as the validation set. Therefore, 11 reference sets (N = 500 for each set) and evaluation sets (N = 50 for each set) were obtained.

### Prediction accuracy and inflation

Three statistical parameters were used to evaluate the performance of each prediction model, namely, observed prediction accuracy, theoretical prediction accuracy, and prediction inflation. To calculate the observed prediction accuracy, data on the 8122 individuals were analysed using the ABLUP model with batch and sex set as fixed effects. The adjusted phenotype (${\mathrm{Y}}_{\mathrm{c}}$) of each bird was obtained by subtracting estimates of the fixed effects from the phenotype. The observed accuracy of the predicted phenotypic values in the validation population was calculated as the Pearson correlation between either the genomic (GEBV) or pedigree-based estimated breeding value (EBV) and the ${\mathrm{Y}}_{\mathrm{c}}$ of the validation individuals ($\widehat{\mathrm{R}}=\mathrm{corr}[\mathrm{GEBV}, {\mathrm{Y}}_{\mathrm{c}}$] or $\widehat{\mathrm{R}}=\mathrm{corr}[\mathrm{EBV}, {\mathrm{Y}}_{\mathrm{c}}$]) averaged across all validation datasets. The theoretical prediction accuracy was based on the prediction error variance (PEV) of the model, where the PEV was estimated by the error of variance between GEBV and the true genetic value (G), as follows [6]:where  is the additive genetic variance. The theoretic accuracy is defined as the correlation between GEBV and G ($\widehat{\mathrm{R}}=\mathrm{corr}[\mathrm{GEBV},\mathrm{ G}$] or $\widehat{\mathrm{R}}=\mathrm{corr}[\mathrm{EBV},\mathrm{ G}$]) without selection and under the assumption that the model is correct. The theoretical accuracy of prediction was estimated using BLUPF90 for the ABLUP model, GVCBLUP for the GBLUP model, and MTG2 for the GFBLUP model. Prediction inflation was calculated as the regression of ${\mathrm{Y}}_{\mathrm{c}}$ on either GEBV or EBV in the validation set. For this statistic, values approaching 1 represent superior results.

### Contribution of QTL to genomic heritability and prediction accuracy

We used two methods to estimate the contribution of QTL to genomic heritability and prediction accuracy: (1) comparison of full and reduced models, (2) estimation of the heritability of two GRM (the significant SNP set from GWAS and the remaining set), and (3) evaluation of the prediction accuracy of the GFBLUP model.

#### Method 1: Comparison of the full and reduced models

The contributions of QTL to genomic heritability and prediction accuracy were defined as “partial heritability” and “partial accuracy”, respectively, based on results from a full model and a reduced model. The GBLUP model that fits all SNPs as random effects was used for the full model. For each run, significant SNPs (P < 3.30e − 7, Bonferroni correction) of the ${\mathrm{i}}$^th^ QTL were selected from the GWAS analysis. Then, the reduced model fitted significant SNPs as fixed effects to remove their effects and those of other SNPs that were in high LD with the targeted SNPs from the random genetic effects on phenotype [30–32]. The GVCBLUP package was used to calculate the genomic heritability and the GEBV. The reduced model was described as:

$${\text{QTL reduced model}}: \mathbf{y}={\mathbf{X}}_{\mathbf{b}}\mathbf{b}+{\mathbf{X}}_{\mathbf{s}}\mathbf{s}+\mathbf{Z}\mathbf{a}+\mathbf{e},$$

where $\mathbf{b}$ is the vector of fixed batch–sex effects; $\mathbf{s}$ is the column vector of fixed SNP effects; $\mathbf{a}$ is the vector of additive genetic values based on genomic information; and ${\mathbf{G}}_{\mathbf{a}}$ is the genomic additive relationship matrix. Variance components were then estimated using the reduced model. The relative contribution of QTL $\mathrm{i}$ to the heritability and observed (theoretical) prediction accuracy was calculated as follows:

$${\mathrm{C}} ({\mathrm{H}}^{2})={({\mathrm{h}}^{2}-{\mathrm{h}}_{\mathrm{i}}^{2})}/{{\mathrm{h}}^{2}},$$

$$\mathrm{C}(\widehat{\mathrm{R}})=(\widehat{\mathrm{R}}-\widehat{{\mathrm{R}}_{\mathrm{i}}})/\widehat{\mathrm{R}},$$

where ${\mathrm{h}}^{2}$ (${\mathrm{h}}_{\mathrm{i}}^{2}$) is the estimated heritability from the full (reduced) model, and $\widehat{\mathrm{R}}$($\widehat{{\mathrm{R}}_{\mathrm{i}}}$) is a measure of prediction accuracy for the full (reduced) model. Both $\widehat{\mathrm{R}}$ and $\widehat{{\mathrm{R}}_{\mathrm{i}}}$ were calculated as the average observed prediction accuracy or average theoretical prediction accuracy.

#### Method 2: Estimating the contribution of two GRM using the GFBLUP model

In this method, which was based on the GWAS results, we split the 151,519 SNPs into a set with the significant GWAS results and a set with the others. Then, we used GCTA to compute a GRM for each set. Using the GRM, the GFBLUP model was used to estimate the additive variance explained by each GRM, and the contribution of the $\mathrm{i}$th QTL region (QTL_i_ on GGA1, GGA2, GGA4, GGA17, or GGA27) to heritability. Observed prediction accuracy was computed as:

$$\mathrm{C}({\mathrm{H}}^{2})={{\mathrm{h}}_{\mathrm{qtl}}^{2}}/({{\mathrm{h}}_{\mathrm{qtl}}^{2}+{\mathrm{h}}_{\mathrm{snp}}^{2}}),$$

$$\mathrm{C}(\widehat{\mathrm{R}})=({\widehat{\mathrm{R}}-\widehat{{\mathrm{R}}_{\mathrm{s}}}})/{{\mathrm{r}}_{\mathrm{all}}},$$

where ${\mathrm{h}}_{\mathrm{qtl}}^{2}$ and ${\mathrm{h}}_{\mathrm{snp}}^{2}$ represent the phenotypic variance explained by the GRM computed from significant and non-significant SNPs, respectively, and $\widehat{\mathrm{R}}$ and $\widehat{{\mathrm{R}}_{\mathrm{s}}}$ represent the observed prediction accuracy computed by both GRM and the GRM composed of non-significant SNPs, respectively. The theoretical prediction accuracy obtained by MTG2 was the square root of the combined reliability (based on PEV of GEBV for each individual) computed by both GRM.

The change in prediction inflation addition for the above two methods was calculated as follows:

$$\mathrm{C}\left(\mathrm{Inflation}\right)=\mathrm{abs}\left({\mathrm{inflation}}_{\mathrm{r}}-1\right)-\mathrm{abs}\left({\mathrm{inflation}}_{\mathrm{all}}-1\right),$$

where the $\mathrm{abs}$ function represents the absolute value between prediction inflation and 1, while ${\mathrm{inflation}}_{\mathrm{r}}$ and ${\mathrm{inflation}}_{\mathrm{all}}$ represent the prediction inflation of the prediction models that used the GRM computed by non-significant SNPs and both GRM, respectively.

### Contribution of SNPs ranked by GWAS

We used the full model described above to estimate the contribution of the GWAS top SNPs (1%, 5%, 10%, 20%, 30%, 40%, 50%, 60%, 70%, 80%, and 90%) by removing each in turn from the statistical model. To avoid data reuse, we repeated a random, tenfold cross-validation GWAS. For each fold validation, 5723 birds (nine partitions of the full set of data) were used for GWAS and reference, and the remaining were used as the validation set. To avoid obtaining SNPs from the same QTL with variants in high LD, we used LD pruning to remove one SNP in each SNP pair with an LD > 0.9 using the PLINK ‘-indep-pairwise’ command [23]. The removed SNPs were not included in the GRM computation for the GBLUP model. As a result, a subset of 118,570 SNPs that tagged all other SNPs with LD ${\mathrm{r}}^{2}$ < 0.9 was obtained. After removing the top SNPs, the remaining tagged SNPs were used to compute the GRM using the GBLUP model of the GVCBLUP package to perform genomic evaluation on the 10th dataset. The relative contribution of each SNP set to the prediction accuracy and heritability was calculated as follows:

$$\mathrm{C}(\widehat{\mathrm{R}})=(\mathrm{r}-{\mathrm{r}}_{90})/r,$$

$$\mathrm{C}({\mathrm{H}}^{2})=({{\mathrm{h}}^{2}-{\mathrm{h}}_{90}^{2}})/{{\mathrm{h}}^{2}},$$

where $\mathrm{r}$ and ${\mathrm{h}}^{2}$ are the measures of prediction accuracy and heritability, respectively, for the full model using all tagged SNPs; ${\mathrm{r}}_{90}$ is the measure of observed prediction accuracy or theoretical prediction accuracy for the GBLUP model based on the GRM construction with tagged SNPs after removing the top SNPs (1–90%); and ${\mathrm{h}}_{90}^{2}$ represents the measure of heritability using tagged SNPs after removing the top SNPs (1–90%). Both $\mathrm{r}$ and ${r}_{90}$ were calculated as the average Pearson correlation between the GEBV and ${\mathrm{Y}}_{\mathrm{c}}$, and were averaged across all validation datasets.

## Results

### QTL identification using GWAS

**Fig. 1:**
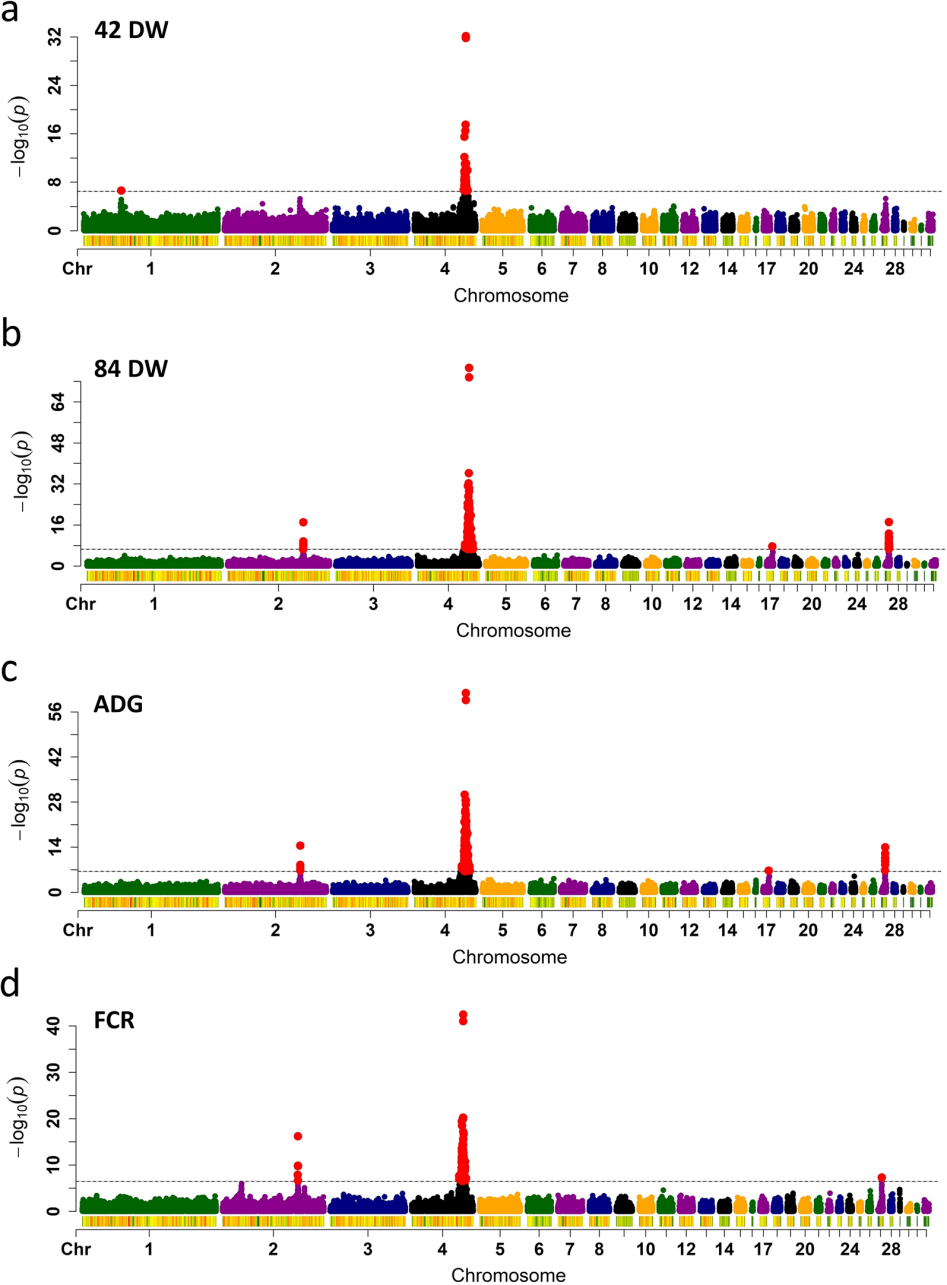
Manhattan plots of the genome-wide association study for: (**a**) 42-day-old weight (DW), (**b**) 84 DW, (**c**) average daily gain (ADG), and (d) feed conversion ratio (FCR). Horizontal grey dashed lines indicate the whole-genome significance threshold (P-value = 3.30e−7)

**Fig. 2:**
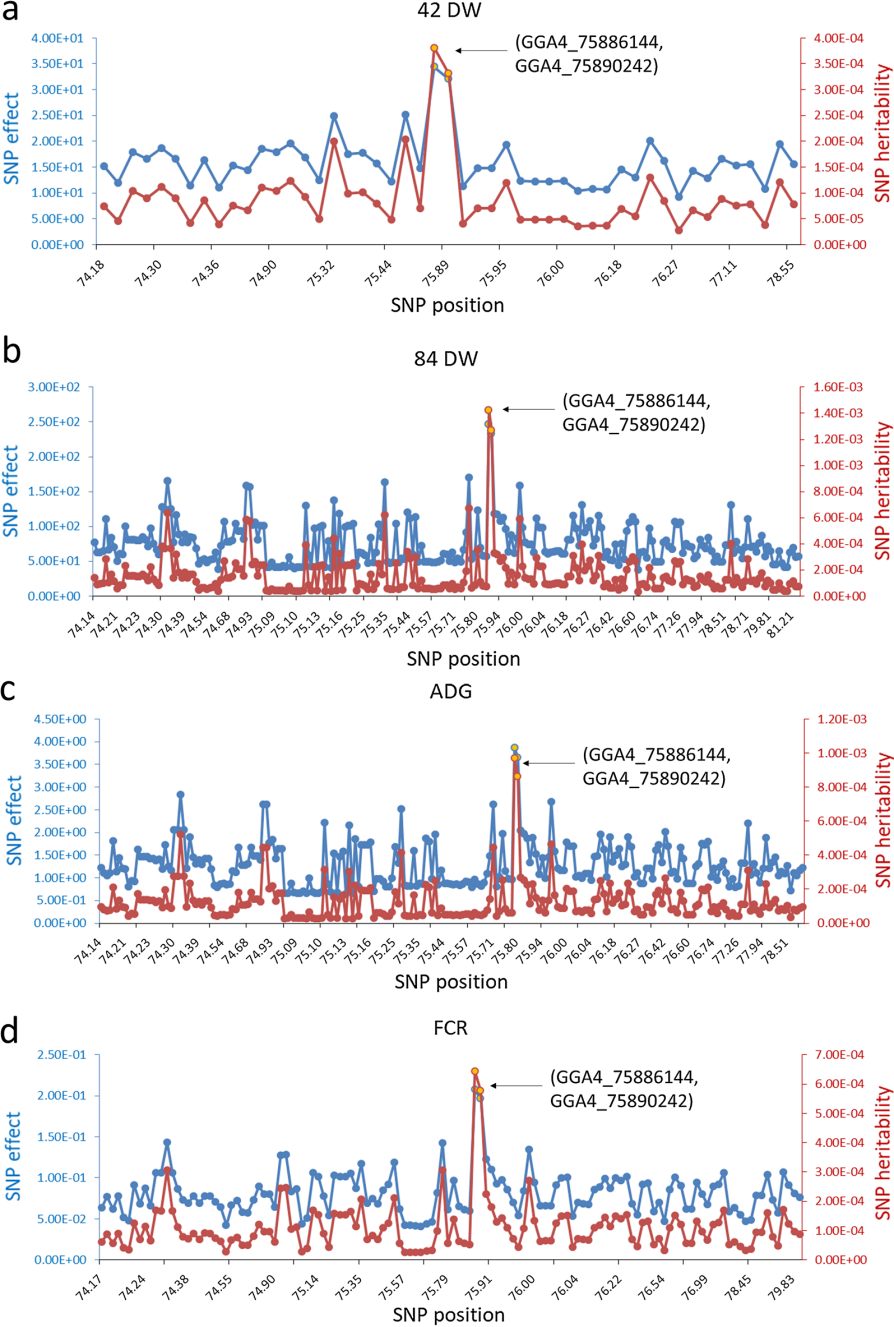
Estimates of additive effects and contributions to heritability of significant SNPs in the QTL region on chromosome 4 for: (**a**) 42-day-old weight (DW), (**b**) 84 DW, (**c**) average daily gain (ADG), and (**d**) feed conversion ratio (FCR). The highest peak emerged at around 75.89 Mb (blue and red lines); yellow dots represent SNPs with the greatest significance (GGA4_75886144 and GGA4_75890242)

The GWAS of four growth traits (42 DW, 84 DW, FCR, and ADG; Table 1) and (see Additional file 1: Figure S1) identified five non-overlapping QTL intervals with SNPs that reached genome-wide significance (Bonferroni multiple testing correction; P < 3.30e−7), which were located on GGA1, 2, 4, 17, and 27, respectively (Fig. 1) and (see Additional file 1: Figure S5). The most significant QTL was located on GGA4, which was associated with all traits, indicating that major genes affecting growth and feed efficiency traits may be located within this region. Two SNPs (GGA4_75886144 and GGA4_75890242) located in an intron of an unknown gene (ENSGALG00000054173) had the most significant P-value for all traits (see Additional file 2: Tables S1–S4), as its significance level far exceeded that of the other SNPs. We also estimated the effects and contribution to heritability of these two SNPs based on the GBLUP model implemented using GVCBLUP [25]. The results showed that these SNPs had the greatest contributions (Fig. 2), suggesting that they may be positioned near the causative genes.

In addition to the QTL on GGA4, we also detected QTL for different traits in different genomic regions. Comparing the GWAS results for 42 DW and 84 DW, the greatest differences were found on GGA1 (QTL detected only for 42 DW), GGA17, and GGA27 (QTL detected only for 84 DW). We also compared the GWAS results for ADG and FCR, which were measured during the same period (birds 42 to 84 days old), and the difference between the detected QTL was identified on GGA17. Collectively, these results indicated that the detected QTL affected chicken growth traits that had significant differences.

### Improvement of prediction accuracy using GBLUP

We performed genomic evaluations with the GBLUP model, using 151,519 SNPs for the four traits. The observed average accuracy of the GEBV from the tenfold validation data set ranged from 0.31 to 0.45 for the GBLUP model (Table 2), which was 22.0 to 70.3% higher than that of the EBV from the same dataset with the pedigree-based ABLUP model (0.25–0.27). The theoretical average accuracy of GBLUP was improved by 26.7 to 45.6% (0.68–0.76) compared to that of ABLUP (0.51–0.57; Table 2). The average prediction inflation of both GEBV and EBV was at a similar level for 42 DW, 84 DW, and ADG, but not for FCR (see Additional file 2: Table S5). The large improvement in prediction was achieved by harnessing genomic information that far exceeded the results of the pedigree method. As expected, the prediction accuracy of the GEBV largely depended on the size of the reference group; that is, the average observed (theoretical) prediction accuracy of GEBV improved with an increase in reference population size, and the prediction inflation of all four growth traits tended to stabilise to 1 (see Additional file 1: Figure S6). The estimate of genomic heritability also had a significant impact on prediction accuracy. The estimated genomic heritability was lowest for 42 DW (Table 2), as was the prediction accuracy. Therefore, a larger reference population is required to increase the predictive ability of 42 DW.

**Table 2:** Estimates of heritability and average prediction accuracy based on tenfold cross-validation for different models

Phenotype	Genomic heritability	ABLUP accuracy	GBLUP accuracy	GFBLUP accuracy
42 DW	0.31	0.26/0.52	0.31/0.69	0.35
84 DW	0.51	0.27/0.52	0.45/0.76	0.52
FCR	0.42	0.26/0.57	0.39/0.72	0.44
ADG	0.42	0.25/0.51	0.42/0.74	0.48

*ABLUP* pedigree-based best linear unbiased prediction, *GBLUP* genomic best linear unbiased prediction, *GFBLUP* genomic feature BLUP

Numbers before and after “/” are the observed prediction accuracy and theoretical prediction accuracy, respectively

### Contributions of QTL to genomic heritability and prediction accuracy

Based on the reduced GBLUP model that fitted significant SNPs within a QTL region as fixed effects, the contribution of a QTL was estimated as the reduction in heritability and prediction accuracy compared to the full model. The QTL on GGA4 contributed the most to all four phenotypes, with the contribution to heritability ranging from 14.5 to 34.5%, and the contribution to the observed and theoretical prediction accuracy ranging from 23.3 to 46.7% and 4.5 to 11.1%, respectively. The contributions to GP and heritability differed between traits. In particular, for 84 DW, the contribution was more than twice as high as that for 42 DW (Table 3) and (see Additional file 2: Table S6), and the prediction inflation for the reduced model was also increased compared to that of the full model for 42 DW (see Additional file 2: Table S7), which indicates that this QTL has a greater effect on late growth and development stages. Although the contributions of the QTL on GGA1 (42 DW), GGA2 (84 DW, ADG, and FCR), GGA17 (84 DW and ADG), and GGA27 (84 DW, ADG, and FCR) were markedly lower than that of the QTL on GGA4, their effects were not negligible. For example, the QTL on GGA1, detected only for 42 DW, contributed 2.6 and 2.3% (0.5%) to heritability and observed (theoretical) prediction accuracy, respectively, suggesting a relatively considerable contribution of this QTL to early growth stages in this population. The contribution of the QTL on GGA2, GGA17, and GGA27 to the observed (theoretical) prediction accuracies were only 3.16%, 0.60%, and 3.58% (0.35%, 0.06%, and 0.25%) for 84 DW, respectively (Table 3) and (see Additional file 2: Table S6), which was consistent with the idea that growth traits are affected by many loci with minor effects [4, 5].

**Table 3:** QTL contributions to heritability and prediction accuracy (%)

QTL region	42 DW	84 DW	FCR	ADG
GGA1: 53.90–56.28 Mb	2.59/2.30	–	–	–
GGA2: 110.08–113.21 Mb	–	1.00/3.16	2.53/4.49	1.04/4.26
GGA4: 74.14–81.87 Mb	14.53/23.30	32.09/46.65	23.87/28.79	34.14/43.35
GGA17: 7.60–9.80 Mb	–	0.43/0.60	–	0.49/0.44
GGA27: 5.91–6.10 Mb	–	0.68/3.58	0.16/0.66	0.34/2.70

Numbers before and after “/” are the contribution to heritability and GP accuracy, respectively

*QTL* quantitative trait loci, *DW* day-old weight, *FCR* feed conversion ratio, *ADG* average daily gain

### Harnessing QTL markers through the GFBLUP model

As described above, the contribution of the detected QTL was considerable, hence, we used a two-GRM GBLUP model (GFBLUP) to improve the prediction accuracy by allowing for increased weight on detected QTL through a separate GRM. As a result, the average accuracy of GP was improved by 13.8 to 15.2% compared to the conventional GBLUP model (Table 2). In addition, the prediction inflation for the GFBLUP model was lower than that of the GBLUP model, and it was maintained at a steady level (0.95 to 0.97) for different growth traits (see Additional file 2: Table S5), indicating that it is important to attribute a greater weight to genetic regions that significantly contribute to phenotypes.

Using the GFBLUP model, we also estimated the genetic variance explained by each GRM and evaluated their predictive ability. We regarded the contribution of the GRM built by significant SNPs in each QTL interval as the representative of each QTL’s contribution. As a result, the contribution of the QTL interval on GGA4 still far exceeded that of the others, with the contribution to heritability ranging from 12.6 to 29.0% and the observed prediction accuracy ranging from 35.0 to 49.5% (see Additional file 2: Table S8). We also observed a relatively minor difference in the theoretical prediction accuracy between the GRM based on significant SNPs (0.51–0.57) and the GRM based on the other SNPs (0.55–0.62) (see Additional file 2: Table S9). In addition, the most marked increase in average prediction inflation was obtained when the effects of the QTL interval on GGA4 were not considered compared to those of the other QTL (see Additional file 2: Table S10). The contributions of the other QTL intervals to the prediction accuracies were also negligible. For example, the contribution of other QTL to observed prediction accuracies was 1.37 to 6.77% for 84 DW and 1.29 to 5.52% for ADG (see Additional file 2: Table S8). This result was generally consistent with that obtained using the GBLUP reduced model, thus demonstrating the major effect of the QTL on GGA4 and the minor effects of the QTL on GGA1, 2, 17, and 27.

## Discussion

### Comparison with QTL from previous studies and candidate genes

Recently, low-cost genotyping analysis using NGS has provided new data resources for genetic population analysis. GBS can provide convenient and sufficient resolution for GWAS and GS studies. In this study, we performed large-scale GBS in a nucleus breeding population of 6359 yellow-plumage chickens. Five significant QTL regions were identified; the most significant QTL, on GGA4, has been widely reported in previous studies. Park et al. detected multiple loci using an F2 AIL population, including the QTL region on GGA4 [2]. Gu et al. identified the same QTL interval, which spanned 8.6 Mb, by performing a GWAS on an F2 chicken resource population derived from a cross between Silky Fowl and White Plymouth Rock [33]. Other studies further narrowed the QTL region to a length of 1.5 Mb, harbouring 15 genes [34, 35]. Although we were unable to further narrow the size of the QTL interval in our study due to a strong level of LD and limited SNP density, we identified two significant SNPs with the highest effects and contribution to heritability of growth traits. The most promising causal genes may be located near these two sites. Hence, further studies should aim at improving the SNP density within this locus.

The QTL region on GGA4 harbours multiple genes. Functional explorations in previous studies have suggested that growth traits may be influenced by multiple genes in this interval. For instance, the *LCORL* and *NCAPG* genes, which have been reported to be associated with body size in humans, cattle, and horses, may have an important influence on growth traits [36]. The *LCORL* gene was also found to be expressed at higher levels in the breast muscle of high-muscle-weight chickens than in low-muscle-weight chickens [37]. The *NCAPG* gene plays a role in growth and development processes in mammals and has been identified as an important candidate gene for growth through the regulation of arginine metabolism [38]. Furthermore, variants in the *SLIT2* gene have been shown to have multiple effects, ranging from an association with spontaneous preterm birth to correlations of its placental expression with human foetal growth [39], and it has been identified as a candidate gene for internal organ weight in beef cattle [40]. In addition, the *LCORL*, *LAP3*, and *FAM184B* genes have been identified as candidate genes for organ weight and body growth in cattle and sheep [40, 41].

Based on a search of the Chicken QTL Database [15], the QTL identified on GGA1, 2, 17, and 27 are also consistent with those identified in several previous studies. The QTL region on GGA1 for 42 DW was identified in two studies; however, they reported different interval lengths, including a region between 45.66 and 54.61 Mb that was associated with body weight at nine days (early growth stage), and a region between 51.21 and 55.21 Mb found to be related to duodenum weight [42]. The QTL on GGA2 was equivalent to a QTL previously identified between 111.52 and 112.37 Mb that was reportedly associated with body weight at different growth stages, including body mass at 28, 56, and 112 days [43]. Although the P-value of the SNPs on GGA2 did not reach significance for 42 DW, a relatively high peak was present for 42 DW in the same region as the QTL on GGA2 that was identified for other growth traits. This result suggests that this QTL may also affect body weight at early developmental stages in this population. In fact, three studies have identified QTL for FCR on GGA17 [44–46], which overlapped with the QTL for body weight identified in our study. The QTL that we identified on GGA27 overlapped with one entry for chicken feed intake in the Animal QTL Database [47], indicating a considerable effect of this QTL interval on chicken growth. These QTL on GGA1, 2, 17, and 27 also harbour genes related to growth traits. For example, the most significant SNP associated with 42 DW on GGA1 was in the intergenic region between the *IGF-1* and *PARPBP* genes. IGF-1 is a well-known factor that is implicated in accelerated growth during childhood and puberty in humans [48]. The IGF-1 pathway is also highly relevant in cattle puberty [49]. This locus may contribute more to early than late developmental stages. The *IGF2BP1* gene is associated with body size and growth in humans, ducks, and goats [50–52] and has been reported as an important candidate gene for fat metabolism and adipogenesis in chickens [37].

### Polygenicity of growth traits in chickens

A previous study has reported that specialised broiler breeds were selected for production traits during long-term domestication, resulting in many growth-related loci that have undergone strong long-term selection, suggesting that growth in broilers is under polygenic control [53]. The influence of growth-related QTL remains to be quantified to evaluate the extent by which they impact growth and genomic evaluation of populations. In this study, we calculated the contribution of each QTL to heritability and genome prediction accuracy using two methods (GBLUP and GFBLUP models); however, the results of these two models differed slightly (Table 3) and (see Additional file 2: Table S8). Using the GBLUP models (full model and reduced model), we included the significant SNPs as fixed factors, which also removed the effects of all SNPs that were in high LD with the fitted SNPs. This may have resulted in the effect of QTL intervals having been overestimated. Therefore, to estimate the contribution of each QTL, we used the GFBLUP model as a comparison with the GBLUP model. Because the estimation of contributions based on the full and reduced GBLUP models (see “Methods”) is affected by the level and extent of LD of the region (Table 3), which were far greater for the QTL on GGA4 than that of the other QTL, the contribution of the GGA4 QTL to heritability was estimated to be larger with GBLUP than with GFBLUP (see Additional file 2: Table S8), and smaller for the other QTL. The estimated contribution of the QTL region on GGA4 to prediction accuracy was, however, larger with the GFBLUP model than with the GBLUP model. This was likely due to the improved performance of the GFBLUP method since the estimated contributions to prediction accuracy of the other QTL were all larger with the GFBLUP model. In general, we conclude that the QTL on GGA4 made the greatest contribution to heritability and prediction accuracy, although the QTL with minor effects that mapped to other chromosomes jointly also made considerable contributions. These results showed that the detected QTL jointly only explained a limited proportion of the genetic variance, which demonstrated the polygenicity of chicken growth traits.

**Fig. 3:**
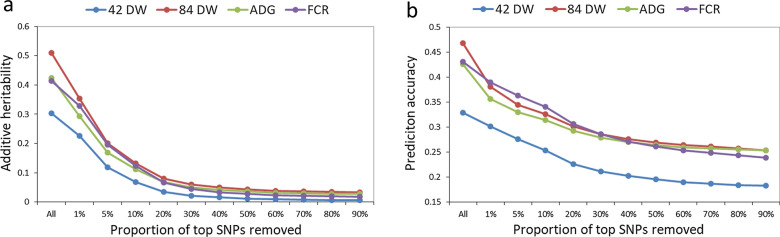
Estimates of the contribution made by the top SNPs to: (**a**) Heritability and (**b**) observed prediction accuracy based on the genomic best linear unbiased prediction (GBLUP) model. DW, day-old weight; ADG, average daily gain; FCR, feed conversion ratio

In addition to significant QTL, other insignificant QTL with smaller effects across the whole genome can also contribute to growth traits [54]. In the current study, many QTL with minor effects could not be detected due to statistical detection limits, however, we were able to evaluate their joint contribution by removing the effects of the top SNPs based on the GWAS P-value (top 1–90%). The estimate of genomic heritability and the observed and theoretical prediction accuracies were drastically reduced when the top 30% of SNPs were removed, resulting in estimates of heritability close to zero for all traits (Fig. 3) and (see Additional file 1: Figure S7). We also observed an increase in the prediction inflation for all traits after removing additional top SNPs from 1 to 90% (see Additional file 1: Figure S7). The results of decreased estimated heritability, decreased prediction accuracy, and increased prediction inflation after removing top SNPs suggest that, besides the major effect of the detectable QTL, there were other undetectable insignificant QTL capable of affecting chicken growth traits that may be more suited to the “infinitesimal model” [55–57]. Finally, we observed that the top 30% of SNPs were widely distributed across the whole genome (see Additional file 1: Figure S8). These results suggest that, in addition to the detectable QTL, a cumulative contribution of SNPs with seemingly insignificant effects also have a considerable influence on the complex traits evaluated here, reflecting the importance of obtaining whole-genome information to improve the accuracy of GP.

### Application of genomic prediction in poultry breeding

Genomic prediction based on genetic markers across the genome has become the standard evaluation method in dairy cattle breeding programmes [8, 58]. In contrast, the shorter generation interval, larger base population, and considerable economic benefits of chickens make them an ideal research subject for GP studies. The traditional poultry breeding method has produced great benefits in recent decades, and GP is expected to further improve upon this conventional breeding method for chickens. In recent years, genomic selection schemes have been applied to poultry breeding [14, 58, 59], and predictive improvement has been continuously studied; however, further studies continue to be greatly needed [11–13]. Although we determined that the GBLUP model significantly improved the accuracy of GEBV, this improvement should be considered as a “proof of principle”. In practice, the cost of genotyping is a major concern. Selection for body weight using the traditional pedigree-based method (ABLUP) has been shown to be highly effective, since the trait has a relatively high heritability and can be measured easily on both sexes and at a young age. To simultaneously control the cost and increase prediction performance, the effect of the size of the reference population should be evaluated when GP is first used in chicken populations, as there is a trade-off between genotyping cost and predictive ability. In our results, we observed a relatively smooth increase in GP accuracy and prediction inflation with a reference population size of 3000–5781 when compared with the trend observed in a population size of 500–3000 (see Additional file 1: Figure S6). Hence, when the reference population reaches a sufficient size, the increase in predictive ability is maintained at a relative gradual level and we can determine the lowest level of reference population size for controlling genotyping cost. In this study, we hypothesise that a reference population size of 3000–4000 is sufficient for maintaining a relatively high level of predictive ability. However, these results were based on random cross-validation and a more stringent evaluation using the parental generation to predict progenies is needed when enough individuals are sequenced. With the rapid decline in genotyping cost, which has thus far exceeded the prediction of Moorer’s Law (https://www.genome.gov/sequencingcostsdata), and with the further advancements in genomic selection for broilers, we expect that large-scale application of whole-genome sequencing will offer improved opportunities for genomic selection in poultry breeding in the near future.

### Pre-selection of markers improves the predictive ability of genomic evaluation

Genomic prediction ability is primarily influenced by the prediction model that is applied [6], the size of the reference population [60], genetic relationships between the reference and validation populations [61], heritability [62], level of LD [63], marker density [64], changes in SNP effects across generations [6], and the complexity of the genetic architecture of the trait (traits that are controlled by a limited number of major genes or complex traits that are influenced by many genes with minor effects) [65].

In our study, we evaluated the contribution of significant SNPs to heritability and prediction accuracy. Figure 3 shows a 74.1 to 81.1% reduction in accuracy after removing the top 30% of significant SNPs based on GWAS. The results indicated that, although each SNP has only a minor effect, the accumulation of the effects of many SNPs throughout the genome has a large impact on prediction accuracy. Although prediction accuracy could be improved by increasing marker density, inclusion of a large number of the sites that do not affect the phenotype could have adverse effects on GP accuracy [66], and preselecting SNPs that contribute to phenotypes can improve prediction accuracy and reduce cost [67–70].

With the increasing availability of whole-genome sequencing data, selected sites that are in high LD with causative variants have become particularly important for estimating the GEBV. In this study, the two-GRM GBLUP model that harnessed preselected significant SNPs improved the prediction accuracy compared to the conventional GBLUP model. However, the preselected SNPs or QTL were confined to a yellow-plumage chicken population, which has not been selected for growth and efficiency to the same extent as commercial broilers. Therefore, the preselected SNPs may not be suitable for assisting GP in commercial broilers. To make the SNP pre-selection method universal, it is important to optimise the method with a more systematic pre-analysis to determine the genetic contribution of each marker required to increase prediction accuracy. For example, for some commercial broilers that have been under long-term selection, there may be no detectable major genes and the genetic architecture of their traits may be more in line with the “infinitesimal model”. In this case, further improvements in GP using only GWAS information may not be feasible. We used GWAS analysis to preselect SNPs and improve prediction accuracy by using the GFBLUP model, however, a more systematic optimisation for preselecting SNPs is needed. Multi-omics meta-analysis provides a promising and accurate strategy for optimising the GP model, since an evaluation that integrates external information may more efficiently and precisely identify loci that have important genetic contributions to phenotypes [71–73]. A recent study in cows that combined functional and evolutionary trait heritability scores and multi-omics information [73] resulted in a set of biological priors for optimising GP and significantly increased prediction accuracy. Overall, pre-selected markers will enable us to determine genomic features more clearly and precisely, as will the further combination of optimised predictive models (such as BayesRC [73]) to improve the performance of GP.

## Conclusions

In this study, we examined the polygenicity and compared the predictive ability of different prediction models for growth traits in a yellow-plumage chicken population: (1) by using GWAS, we identified five candidate QTL intervals for growth traits and confirmed a major effect of the QTL on GGA4 and four other QTL with minor effects on GGA1, GGA2, GGA17, and GGA27; (2) by using the GBLUP model, the prediction accuracy was greatly improved compared to the conventional pedigree-based method (ABLUP model); (3) by accounting for QTL markers using the GFBLUP model, the prediction accuracy was further improved compared to the GBLUP model. Although our genomic evaluation and the genomic feature-harnessing model results demonstrated an improvement of prediction performance, these improvements must be considered as a “proof of principle” for evaluating the feasibility of applying genomic selection in poultry breeding.

## Supplementary Information

**Additional file 1: Figure S1.** Phenotypic distribution of four traits in yellow-plumage chickens (males and females). The data present the phenotypic distribution of four growth traits according to males (left panel) and females (right panel). **Figure S2.** SNP density of 151,519 variants. The data present the SNP density of detected SNPs within 1-Mb windows. **Figure S3.** Principal component analysis (PCA) of birds. PCA of sequenced individuals, where the top two principal components were plotted. **Figure S4.** Study design for cross-validation. The discovery sets were randomly selected from 6359 birds; 1500 individuals were used for GWAS; and the remaining 4859 birds were used for predictive evaluation (ABLUP, GBLUP and GFBLUP models used for tenfold cross-validation), where the accuracy and inflation of prediction were averaged across all validation sets. **Figure S5.** QQ plots of the genome-wide association study of the four traits. The red dots represent SNPs that passed the significance level threshold (P-value < 3.30e^−7^). **Figure S6.** Trend line of genomic prediction accuracy and inflation based on different sizes of the reference population (500 to 5500). Three indicators were used for evaluation, namely (a) observed prediction accuracy, (b) theoretical prediction accuracy, and (c) prediction inflation. **Figure S7.** Trend line of genomic prediction accuracy and inflation. The line is based on the proportion of top SNPs removed based on the GWAS results. (a) Theoretical prediction accuracy, and (b) prediction inflation. **Figure S8.** Genome-wide distribution of the top 30% of SNPs. The distribution of SNPs is based on GWAS results overlapping in the tenfold validation analysis, where the number of overlapping SNPs was equal to 15,077, 14,460, 14,558, and 14,259 in the analysis of 42 DW, 84 DW, ADG, and FCR, respectively.

**Additional file 2: Table S1.** Information on the significant SNPs associated with 42 DW in the QTL region on chromosome 4. The data present the genomic location, effect, heritability, P value, and functional annotation of each GWAS significant SNP associated with 42 DW on chromosome 4. **T**a**ble S2.** Information on the significant SNPs associated with 84 DW in the QTL region on chromosome 4. The data present the genomic location, effect, heritability, P value, and functional annotation of each GWAS significant SNP associated with 84 DW on chromosome 4. **Table S3.** Information on the significant SNPs associated with ADG in the QTL region on chromosome 4. The data present the genomic location, effect, heritability, P value, and functional annotation of each GWAS significant SNP associated with ADG on chromosome 4. **Table S4.** Information on the significant SNPs associated with FCR in the QTL region on chromosome 4. The data present the genomic location, effect, heritability, P value, and functional annotation of each GWAS significant SNP associated with FCR on chromosome 4. **Table S5.** Average prediction inflation based on tenfold cross-validation. The data provide the average prediction inflation using ABLUP, GBLUP or GFBLUP models. **Table S6.** QTL contributions to the theoretical prediction accuracy (%). Description: The contributions were estimated by setting GWAS significant SNPs in each QTL as fixed factors. **Table S7.** Average prediction inflation addition after setting GWAS significant SNPs as fixed factors. The average prediction inflation changes after setting GWAS significant SNPs as fixed factors, where values approaching 0 represent lower inflations. **Table S8.** QTL contributions to heritability and observed prediction accuracy (%) using the GFBLUP model. The contributions were evaluated by estimating the contribution of two GRM using the GFBLUP model. **Table S9.** Average theoretical prediction accuracy using two GRM in the GFBLUP model. The data provide the average theoretical accuracy of prediction based on the GRM of GWAS significant sites and the remaining sites, respectively. **Table S10.** Average prediction inflation addition in the GFBLUP model. The changes in average prediction inflation evaluated by using the GRM without GWAS significant SNPs compared to that using both GRM, where values approaching 0 represent lower inflations.

## Data Availability

The datasets supporting the results of this article are included within the article and its additional files. The phenotypic data and original SNP data are available from the corresponding author upon reasonable request.
