# Supplementary material for: Genome‑wide association study and genomic prediction for growth traits in yellow-plumage chicken using genotyping-by-sequencing
Source: Genet Sel Evol. 2021 Oct 27;53:82. doi: 10.1186/s12711-021-00672-9 (PMC8555081; doi:10.1186/s12711-021-00672-9)
Supplement: Supplementary file 1 — Additional file 1: Figure S1. Phenotypic distribution of four traits in yellow-plumage chickens (males and females). The data present the phenotypic distribution of four growth traits according to males (left panel) and females (right panel). Figure S2. SNP density of 151,519 variants. The data present the SNP density of detected SNPs within 1-Mb windows. Figure S3. Principal component analysis (PCA) of birds. PCA of sequenced individuals, where the top two principal components were plotted. Figure S4. Study design for cross-validation. The discovery sets were randomly selected from 6359 birds; 1500 individuals were used for GWAS; and the remaining 4859 birds were used for predictive evaluation (ABLUP, GBLUP and GFBLUP models used for tenfold cross-validation), where the accuracy and inflation of prediction were averaged across all validation sets. Figure S5. QQ plots of the genome-wide association study of the four traits. The red dots represent SNPs that passed the significance level threshold (P-value < 3.30e−7). Figure S6. Trend line of genomic prediction accuracy and inflation based on different sizes of the reference population (500 to 5500). Three indicators were used for evaluation, namely (a) observed prediction accuracy, (b) theoretical prediction accuracy, and (c) prediction inflation. Figure S7. Trend line of genomic prediction accuracy and inflation. The line is based on the proportion of top SNPs removed based on the GWAS results. (a) Theoretical prediction accuracy, and (b) prediction inflation. Figure S8. Genome-wide distribution of the top 30% of SNPs. The distribution of SNPs is based on GWAS results overlapping in the tenfold validation analysis, where the number of overlapping SNPs was equal to 15,077, 14,460, 14,558, and 14,259 in the analysis of 42 DW, 84 DW, ADG, and FCR, respectively. [file 12711_2021_672_MOESM1_ESM.docx]

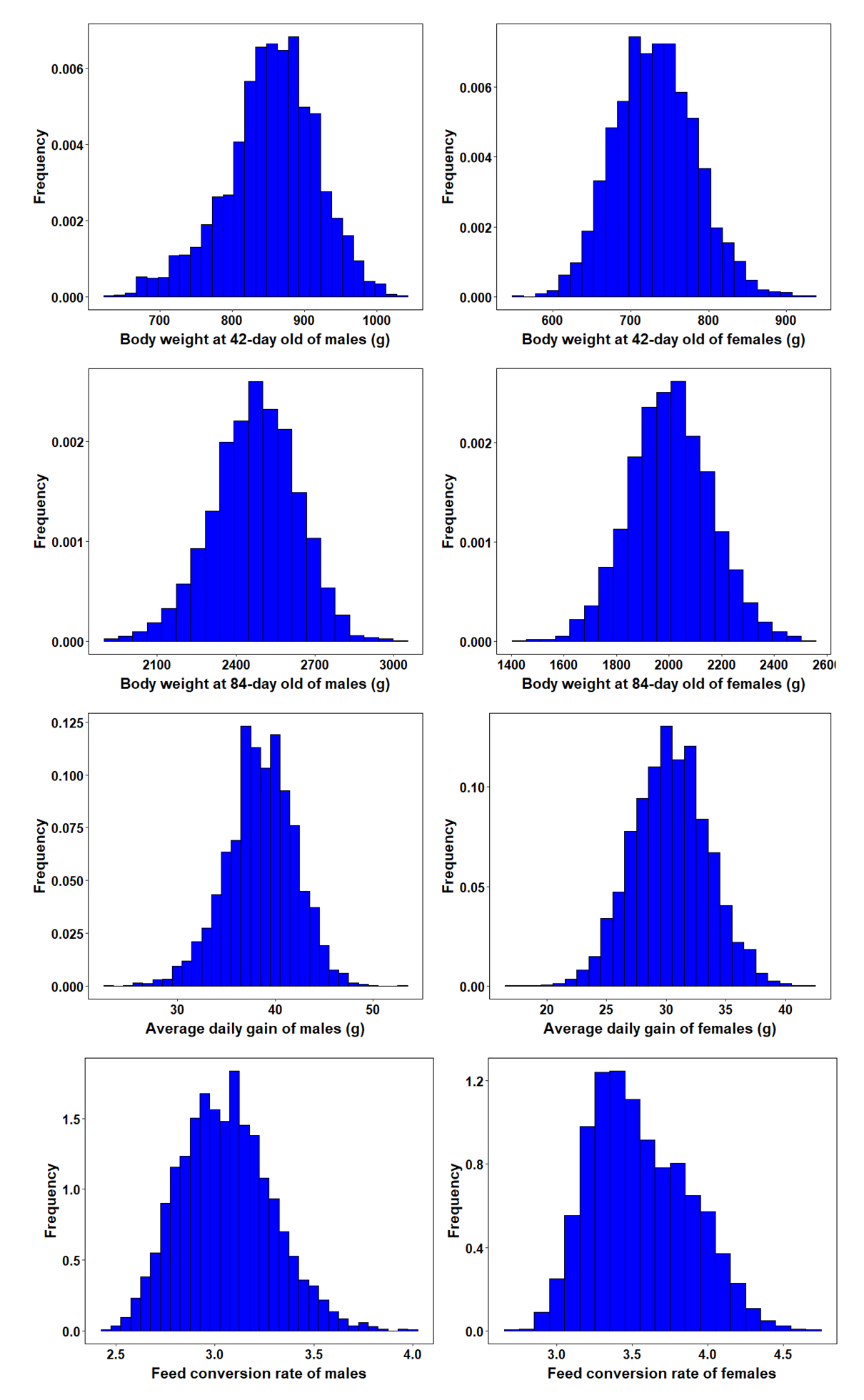


**Figure S1.** Phenotypic distribution of four traits in yellow-plumage chickens (males and females).


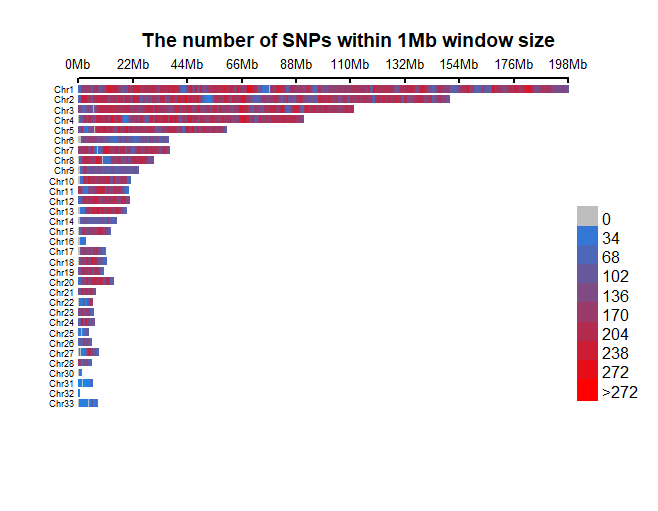


**Figure S2.** SNP density of 151,519 variants.


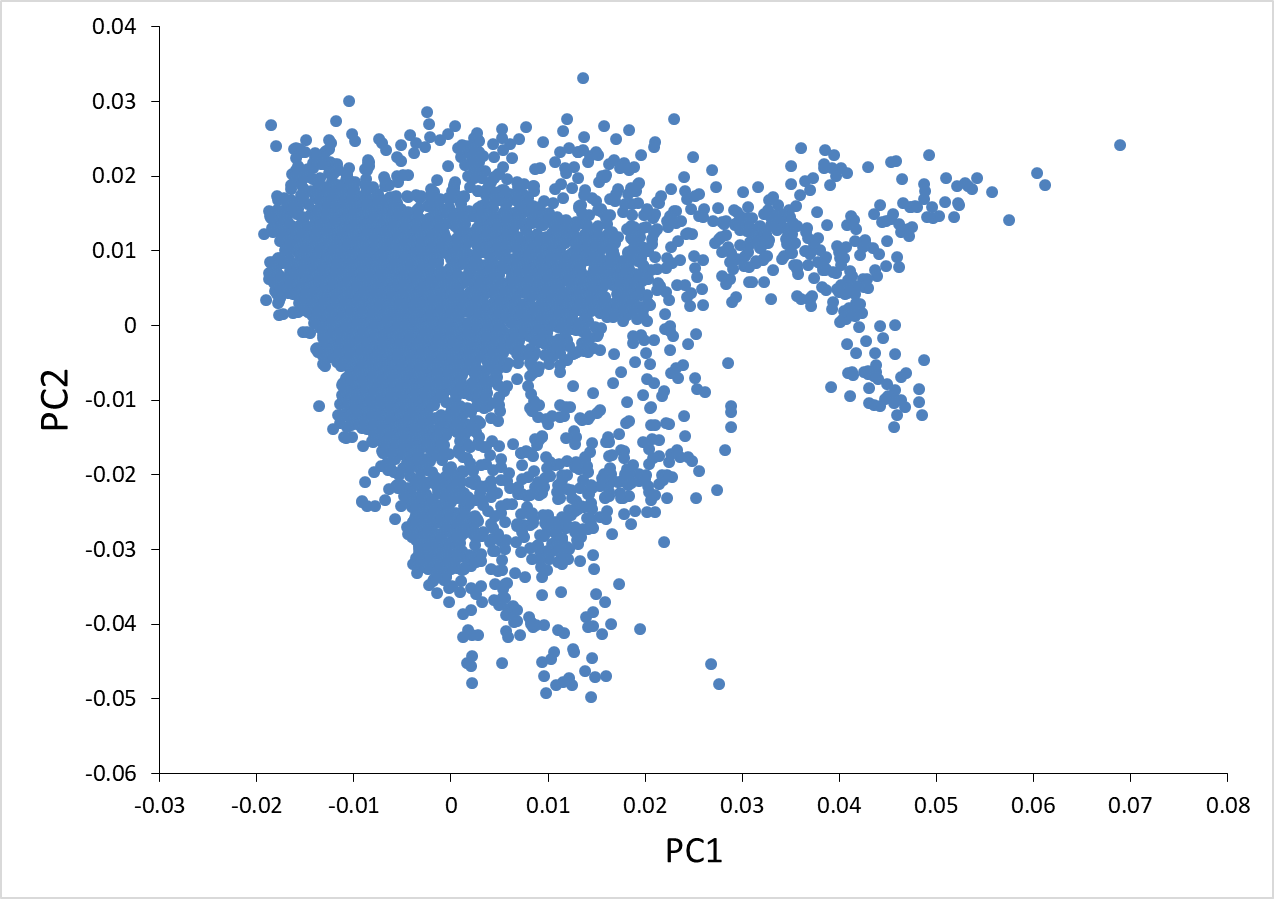


**Figure S3.** Principal component analysis (PCA) of birds.


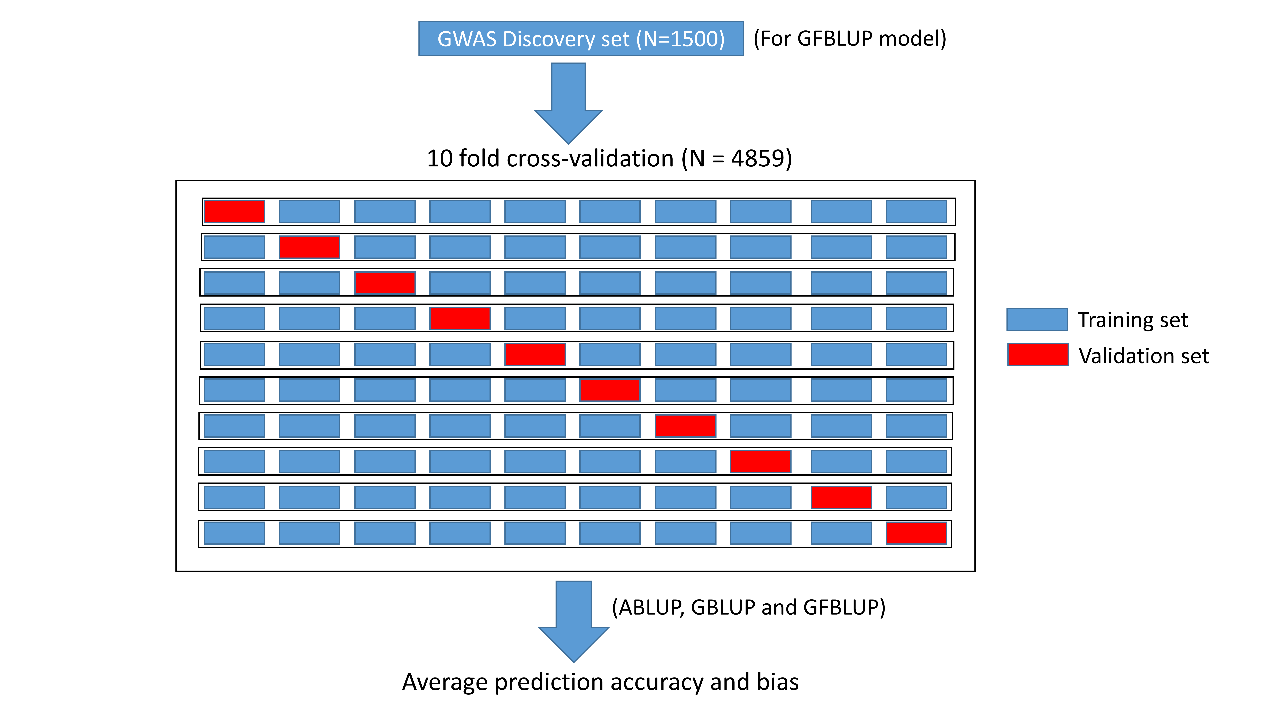


**Figure S4.** Study design for cross-validation.


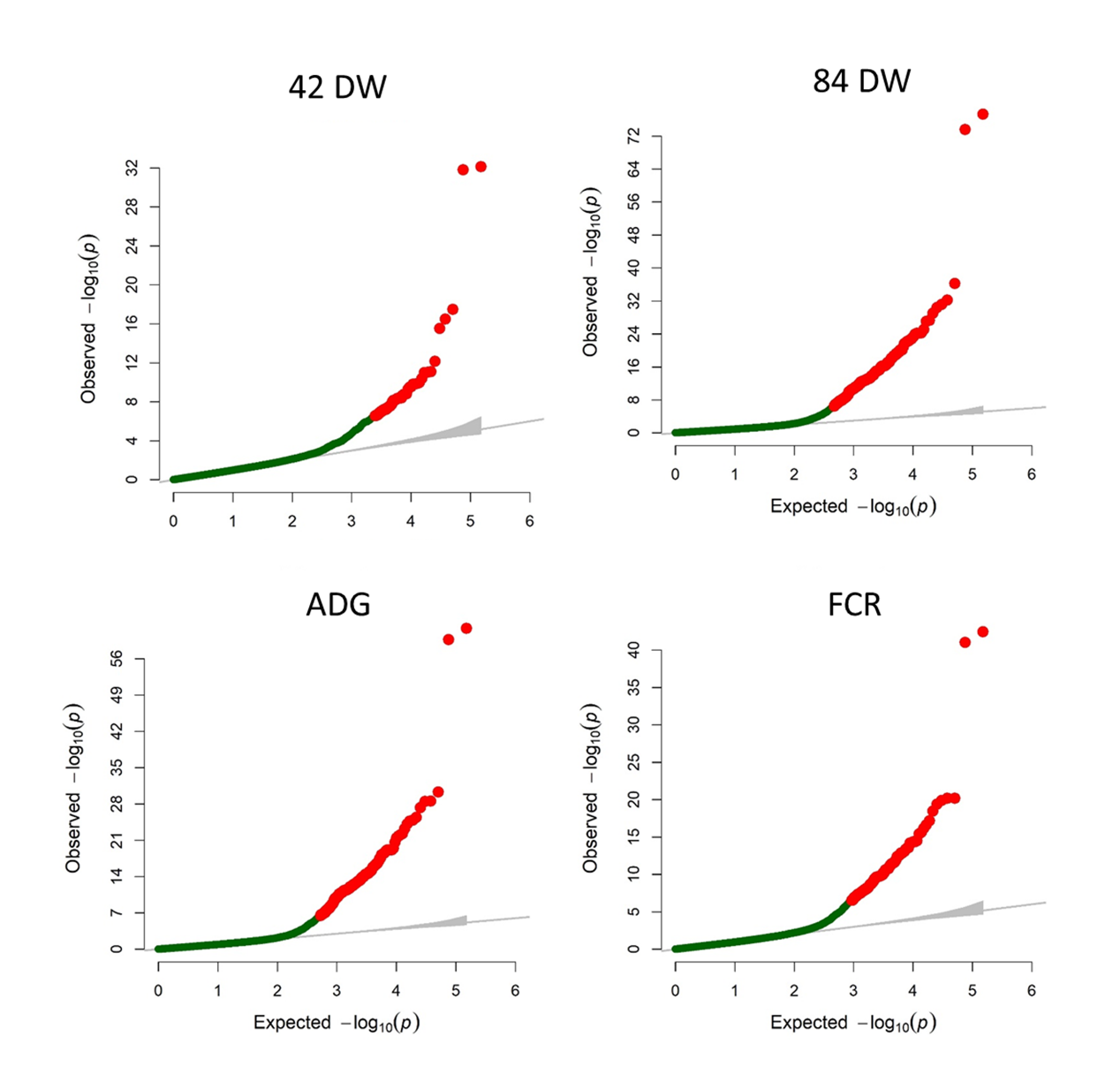


**Figure S5.** QQ plots of the genome-wide association study of the four traits.


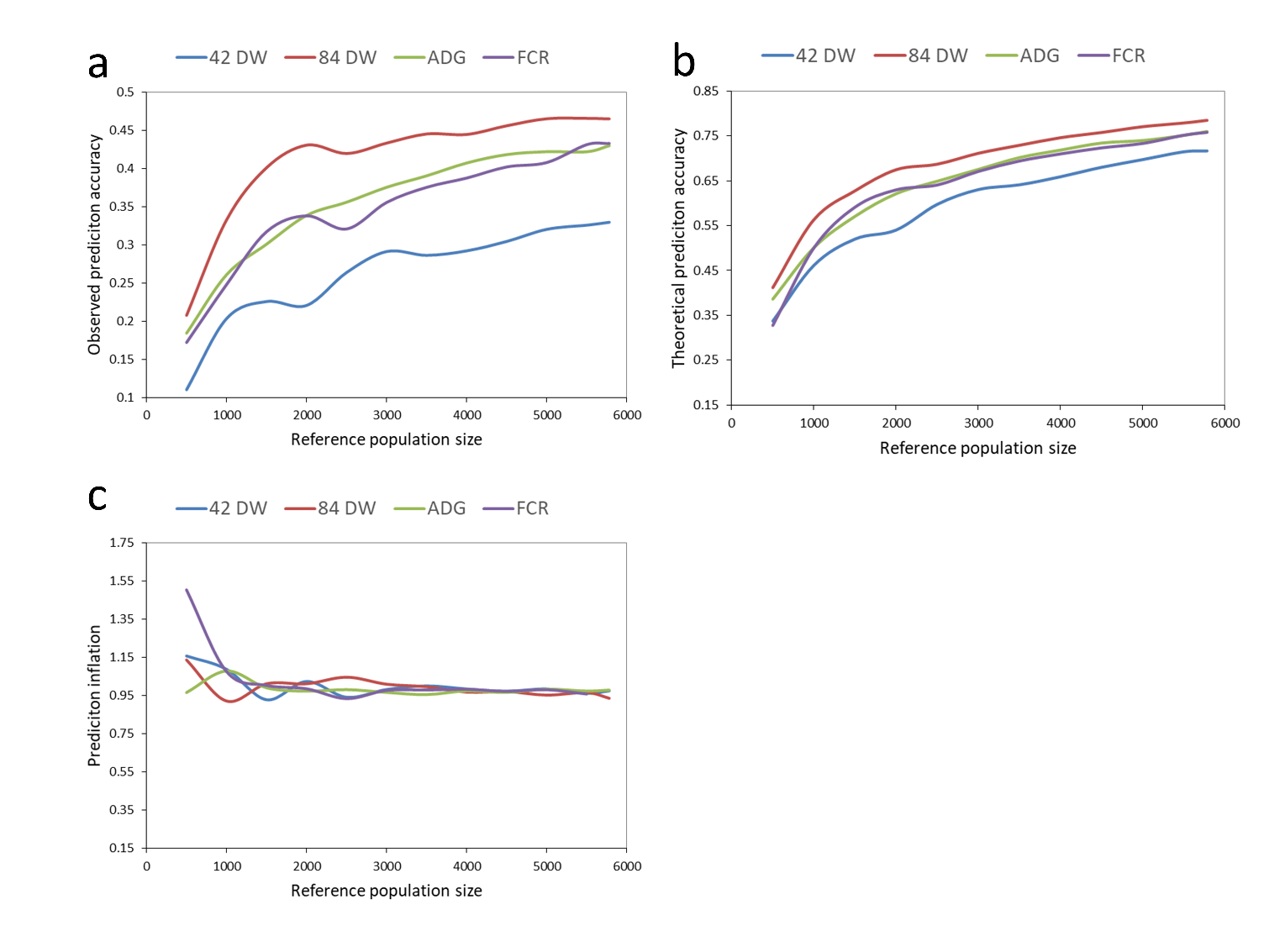


**Figure S6.** Trend line of genomic prediction accuracy and inflation based on different sizes of the reference population (500–5500).


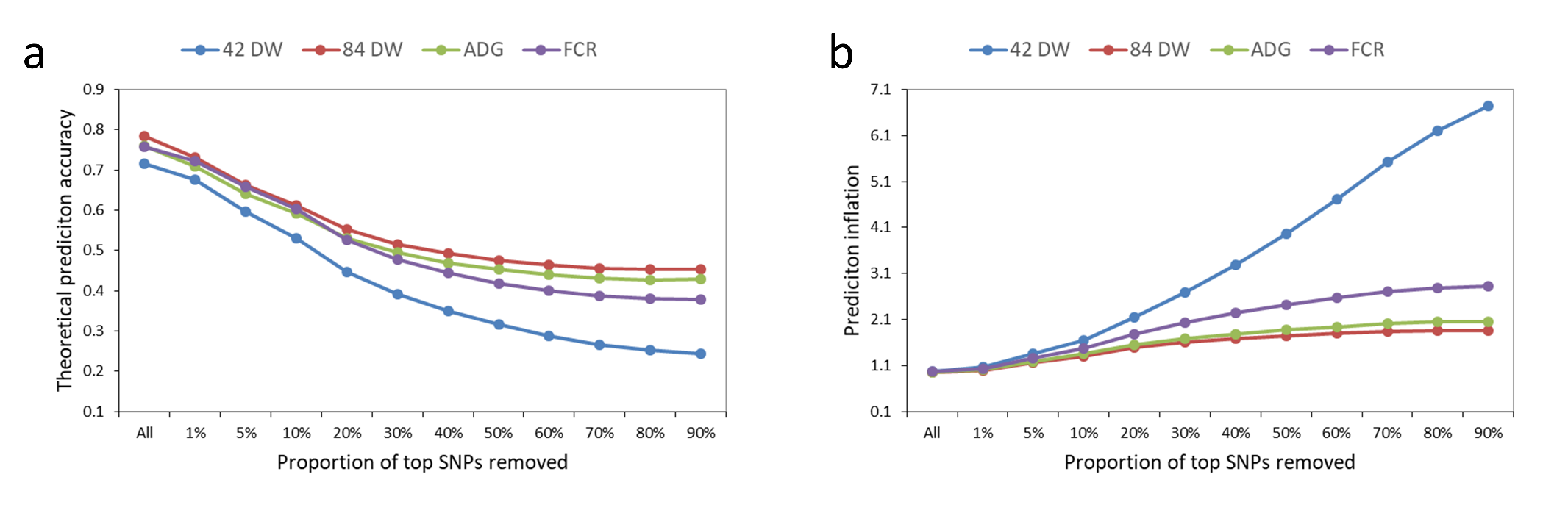


**Figure S7.** Trend line of genomic prediction accuracy and inflation.


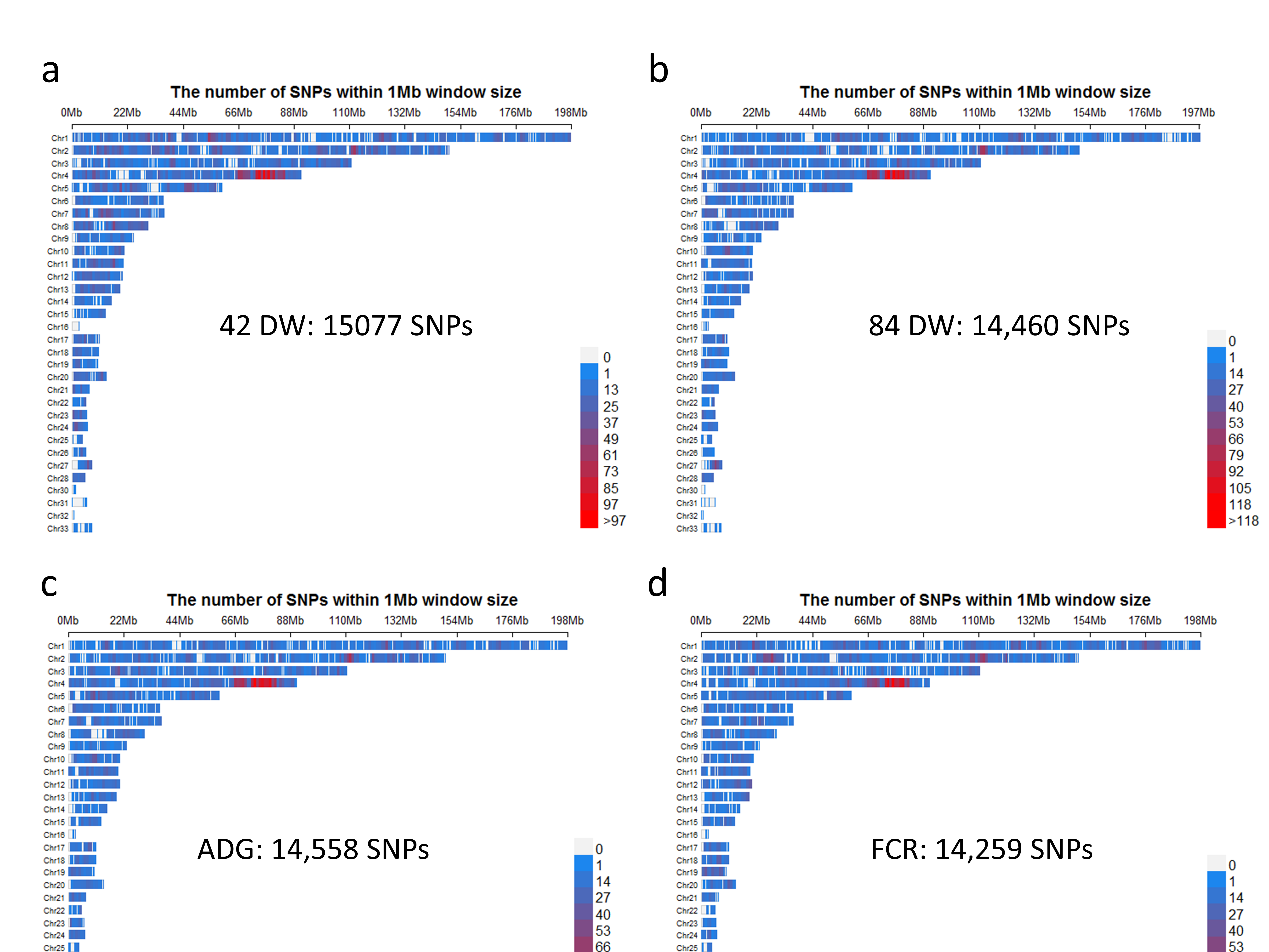


**Figure S8.** Genome-wide distribution of the top 30% of SNPs.
